# N-Glycans and Glycosylphosphatidylinositol-Anchor Act on Polarized Sorting of Mouse PrP^C^ in Madin-Darby Canine Kidney Cells

**DOI:** 10.1371/journal.pone.0024624

**Published:** 2011-09-08

**Authors:** Berta Puig, Hermann C. Altmeppen, Dana Thurm, Markus Geissen, Catharina Conrad, Thomas Braulke, Markus Glatzel

**Affiliations:** 1 Institute of Neuropathology, University Medical Center Hamburg-Eppendorf, Hamburg, Germany; 2 Department of Biochemistry, Children's Hospital University Medical Center Hamburg-Eppendorf, Hamburg, Germany; University of Melbourne, Australia

## Abstract

The cellular prion protein (PrP^C^) plays a fundamental role in prion disease. PrP^C^ is a glycosylphosphatidylinositol (GPI)-anchored protein with two variably occupied N-glycosylation sites. In general, GPI-anchor and N-glycosylation direct proteins to apical membranes in polarized cells whereas the majority of mouse PrP^C^ is found in basolateral membranes in polarized Madin-Darby canine kidney (MDCK) cells. In this study we have mutated the first, the second, and both N-glycosylation sites of PrP^C^ and also replaced the GPI-anchor of PrP^C^ by the Thy-1 GPI-anchor in order to investigate the role of these signals in sorting of PrP^C^ in MDCK cells. Cell surface biotinylation experiments and confocal microscopy showed that lack of one N-linked oligosaccharide leads to loss of polarized sorting of PrP^C^. Exchange of the PrP^C^ GPI-anchor for the one of Thy-1 redirects PrP^C^ to the apical membrane. In conclusion, both N-glycosylation and GPI-anchor act on polarized sorting of PrP^C^, with the GPI-anchor being dominant over N-glycans.

## Introduction

Prion diseases, occurring in humans and a wide range of animals, are believed to be caused by misfolding of PrP^C^ into a disease-associated form, PrP^Sc^ [1], [2]. PrP^Sc^ is enriched in β-sheets and forms partially protease-resistant aggregates which mainly accumulate in the central nervous system [3].

A multitude of putative physiological functions have been attributed to PrP^C^ including control of synaptic activity, neuroprotection, neurogenesis (reviewed in [4]), maintenance of myelination [5] or acting as a receptor for β-amyloid oligomers [6]. Interestingly, although PrP^C^ is largely conserved between vertebrates, PrP^C^-deficient mice only show subtle phenotypes [5], [7], [8].

PrP^C^ is a glycosylphosphatidylinositol (GPI)-anchored protein residing in detergent-resistant membranes (DRMs) and removed from DRMs in order to be internalized via clathrin-coated endocytosis. DRMs have been postulated as sites of conversion from PrP^C^ to PrP^Sc^ either directly at the cell surface or in the early endocytic pathway [9]. In addition, divergence or absence of GPI-anchorage of PrP^C^ influences development of prion disease [10], [11].

PrP^C^ is a glycoprotein of 253 amino acids in humans and 254 amino acids in mice that contains two N-glycosylation sites at Asn^181^ and Asn^197^ in humans and Asn^180^ and Asn^196^ in mice. These sites are variably occupied giving rise to the typical electrophoretic mobility pattern of di-, mono-, and non-glycosylated polypeptides [12], [13], [14]. The biological significance of this complex pattern of glycosylation is not known but mutations in the consensus sites for glycosylation lead to genetic forms of Creutzfeldt-Jakob Disease [15], [16].

Polarized cells such as neurons or epithelial cells consist of two specialized plasma membrane domains, the apical and basolateral membranes. The maintenance of polarity and cellular function requires distinct differential protein sorting mechanisms and various signal structures are needed for the selective transport of membrane proteins to the apical or basolateral membranes. In general, N-glycosylated and GPI-anchored proteins are apically sorted when expressed in Madin-Darby canine kidney (MDCK) epithelial cells. The GPI-anchor can act as an apical signal that is well conserved among species [17] and chimeric GPI-anchored proteins are found in the apical compartment [18], [19]. However, addition of the GPI-anchor of T-cadherin to EGFP proved to be insufficient for apical delivery in MDCK cells [20]. The unpolarized delivery of GPI-anchored rat growth hormone fusion protein, could be directed to the apical compartment by the addition of N-glycans [21] and addition of N-glycans to an otherwise unpolarized secreted protein directs it to the apical compartment [22]. Furthermore, mutation of the N-glycosylation sites of the GPI-anchored membrane dipeptidase protein (MDP) resulted in basolateral targeting [23]. Oligomerization appears to form an additional structural element for the sorting of GPI-anchored proteins to the apical side [24], [25].

PrP^C^ is an exception because it is the only N-glycosylated, GPI-anchored protein known to date that is basolaterally sorted in MDCK cells [26]. Signals that regulate basolateral sorting of PrP^C^ are not fully understood but elimination or mutations of the hydrophobic core of PrP^C^ lead to apical sorting [27], suggesting sorting determinants in the luminal domain. In contrast, the transfer of the GPI-anchor signal sequence of PrP^C^ to EGFP resulted in basolateral targeting of the EGFP fusion protein [28].

Because the role of glycosylation in sorting of PrP^C^ is poorly understood, in this study we investigated the role of N-glycans and the GPI-anchor as potential polar sorting signals of PrP^C^ expressed in MDCK cells. The most striking phenotype was that the loss of a single N-glycosylation site resulted in sorting to membranes in an unpolarized manner. In addition, the substitution of the PrP^C^-GPI-anchor by the Thy-1-GPI-anchor, which targets Thy-1 to the apical compartment, redirected PrP^C^ to the apical side. These data suggest that the GPI-anchor represents a dominant basolateral sorting signal of PrP^C^ which can be modulated by N-linked oligosaccharides.

## Materials and Methods

### cDNA constructs

The cDNA containing the mouse *Prnp* open reading frame with the 3F4 mAb epitope tag in pcDNA3.1(+)/Zeo expression vector was a gift from M. Groschup (Institute for Novel and Emerging Infectious Diseases at the Friedrich-Loeffler-Institut, Greifswald - Insel Riems, Germany). Mutations eliminating the consensus site for N-glycans were made with the QuickChange Site-Directed Mutagenesis Kit (Stratagene). For the mutation N180Q, the following primers were used: 5′GTGCACGACTGCGTC**C**A**A**ATCACCATCAAGCAG 3′ (sense) and 5′CTGC TTGATGGTGAT**T**T**G**GACGCAGTCGTGCAC 3′ (antisense). For the N196Q mutation, the following primers were used: 5′GACCACCAAGGGGGAG**C**A**A**TTCACCGAGACCGATG 3′ (sense) and 5′CATCGGTCTCGGTGAA **T**T**G**CTCCCCCTTGGTGGC 3′ (antisense, mutations are in bold). For PrP^C^-GPIThy-1, a fusion PCR approach was used. Thy-1 full length cDNA clone (IMAGENES) was subcloned into pCDNA 3.1(−)/Neo expression vector (Invitrogen). The primers used for the fusion PCR were: for the PrP^C^ moiety, primer A (sense) 5′ACCAGGGATAGCTGCGTTTA 3′ and primer B (antisense) GCCGCCGGATCTT CTCCCGTC and for the Thy-1 moiety, primer C (sense) 5′GATCCGGCGGCATAAGCCTG 3′ and primer D (antisense) 5′AAGCTTAGTTCAGGGCCCCAG 3′. The resulting DNA was inserted into pcDNA3(−)/Neo expression vector (Invitrogen) and all sequences were verified by DNA sequencing.

*Cell culture and transfections*. MDCK cells [29] were grown in Dulbecco's modified Eagle's medium high glucose with L-gutamine, supplemented with 10% fetal bovine serum, penicillin/streptomycin (PAA Laboratories) and 25 mM HEPES (Invitrogen) in a 5% CO_2_ incubator. Transfections were made with Lipofectamine 2000 (Invitrogen) as described by the supplier and after three weeks under selection media (Zeocin 400 µg/ml (Invitrogen) or G418 800 µg/ml (PAA Laboratories)) resistant clones were selected.

### DRMs isolation

Confluent cells plated in a 100 mm Petri dish were washed twice with cold PBS (10 mM phosphate buffered saline pH 7.4) and scraped in TNE buffer (50 mM Tris-HCl, 150 mM NaCl, 2 mM EDTA, pH 7.4.) with 1% Triton X-100 and EDTA-free protease inhibitor cocktail (Roche). Cells were disrupted with a 26G needle and incubated for 30 min in an orbital rotor at 4°C. After centrifugation for 5 min at 500 *g*, supernatants were mixed with OPTIPREP (Sigma), to get a final concentration of iodixanol of 40% and placed in the bottom of a centrifuge tube (UltraClear, Beckmann). 7.5 ml of 30% iodixanol prepared in TNE buffer and 3.5 ml of 5% iodixanol were sequentially layered on top. After 18 h centrifugation at 155.000 *g* in an SW40 Ti rotor (L-60 ultracentrifuge, Beckman), 1 ml fractions were taken from the top. 300 µl of each fraction were acetone precipitated, mixed with 4× sample buffer (250 mM Tris-HCl, 8% SDS, 40% glycerol, 20% β-mercaptoethanol, 0.008% Bromophenol Blue, pH 6.8) and analysed by western blot. The 3F4 anti-mouse antibody (Covance) was used at a dilution of 1∶1,000 [30] and flotillin anti-mouse antibody (BD Transduction) was used at a dilution of 1∶5,000.

### Cell surface biotinylation assays

MDCK cells stably expressing the indicated constructs were plated at density of 2×10^5^ in 24 mm polycarbonate 0.4 µm pore Transwell filters (Costar) and grown for 4 to 5 days until polarization was achieved. Media was changed every other day. To evaluate integrity of the monolayer we used the method described by Lipschutz et al [31], whereby leakiness of the apical fluid is assessed by observation for 12 to 18 hours. Only in instances where leakiness of the apical fluid could be excluded, cells were used for further experiments. For the cell surface biotinylation assay, polarized cells were washed three times with cold Dulbecco's PBS (DPBS, Sigma-Aldrich) containing CaCl_2_ and MgCl_2_ (used in all the experiments) and incubated either apically or basolaterally with EZ-Link Sulfo-NHS-SS-Biotin (Thermo Scientific) in DPBS for 30 min at 4°C while shaking. The reaction was stopped by adding Quenching Solution (Pierce) and extensively washed with TBS (10 mM Tris-HCl pH 7.4, 140 mM NaCl). Membranes were then excised and placed in 1.5 ml tubes containing 500 µl of lysis buffer (25 mM Tetra-Ethyl-Ammonium-chloride (TEA.Cl) pH 8.1, containing 2.5 mM EDTA, 50 mM NaCl, 0.25% SDS and 1.25% Triton X-100) with EDTA-free protease inhibitor cocktail (Roche). After incubation for 1 h at 4°C, samples were centrifuged at 12,000 *g* for 5 min and supernatants were further incubated with High Capacity Neutravidin Agarose beads (Thermo Scientific) in Spin Columns (Pierce) for 1 h at room temperature. After washing extensively with wash buffer (20 mM TEA.Cl pH 8.6, containing 150 mM NaCl, 5 mM EDTA, 1% Triton-X100 and 0.2% SDS) followed by washes with TBS wash buffer without detergents, proteins were eluted by boiling for 5 min with 4× sample buffer. Following electrophoresis, Western blots were incubated with 3F4 antibody as described above and anti-mouse E-Cadherin (BD Transduction) at a dilution of 1∶5,000. After washing with TBST (TBS containing 0.1% Tween-20), secondary anti-mouse or anti-rabbit antibodies (Promega) were used at a dilution of 1∶1,000. Blots were developed with SuperSignal West Pico or West Femto Chemiluminiscent Substrate (Thermo Scientific) in a CD camera imaging system (BioRad). Quantification of at least three independent experiments was made by using Quantity One analysis software (BioRad).

### Confocal immunofluorescence microscopy

Cells plated in 12 mm polycarbonate Transwell filters for 4 to 5 days were placed on ice and washed twice with cold DPBS, incubated with the 3F4 anti-mouse antibody at a dilution of 1∶100 in DPBS with 2% of normal donkey serum (Dianova). After 20 min incubation at 4°C, cells were washed three times in cold DPBS and incubated for 20 min with secondary donkey anti-mouse antibody AlexaFluor488 (Invitrogen) containing 2% of normal donkey serum at 4°C. After three washes with cold DPBS, cells were fixed with 4% paraformaldehyde in PBS for 10 min at room temperature and extensively washed. DAPI (Roche) was added in the last wash and incubated for 5 min in order to visualize nuclei. Filters were cut out and placed cell side up in a microscope slide containing a drop of Fluoromount G (SouthernBiotech) mounting media. For double-immunocytochemistry with 3F4 anti-mouse antibody and rabbit anti-ZO-1 antibody (Invitrogen), the procedure described above for the 3F4 antibody staining was performed first. Then, after fixation with paraformaldehyde and washes with DPBS, cells were incubated for 10 min with DBPS containing 0.1% Triton X-100. Washes between the incubation with primary and secondary antibodies were also performed with DPBS containing 0.1% Triton X-100. Cells were incubated with ZO-1 antibody, used at a dilution of 1∶100 in DPBS containing 2% of normal donkey serum, for 20 min at room temperature. After washing with DPBS containing 0.1% Triton X-100, secondary donkey anti-rabbit antibody AlexaFluor555 (Invitrogen) was diluted in DPBS containing 2% of normal donkey serum and incubated for 20 min at room temperature. After extensive washing with DPBS, DAPI was added in the last wash and samples were mounted as described before. For 3F4 antibody staining under permeabilizing conditions, the same procedure as used for the ZO-1 staining was performed. Consecutive Z-stacks were taken with Leica Laser Scanner Confocal Microscope TCS SP2 (Leica) and images were further processed with the Volocity 5 Software (Perkin Elmer).

## Results

### Lack of glycosylation does not alter plasma membrane localization of PrP^C^

**Figure 1:**
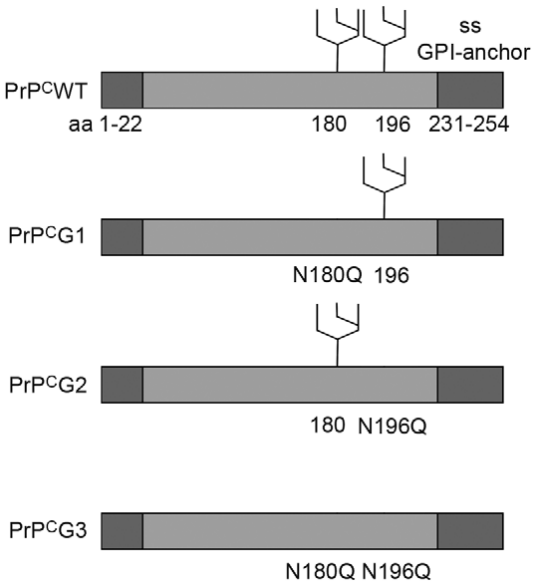
Schematic drawing of constructs used in this study. Shown are the maps of PrP^C^WT, PrP^C^G1, PrP^C^G2, and PrP^C^G3 with N-terminal signal sequence (ss) and C-terminal GPI-anchor signal (ss GPI-anchor) (dark boxes) and the mutations introduced to delete N-gylcosylation sites.

**Figure 2:**
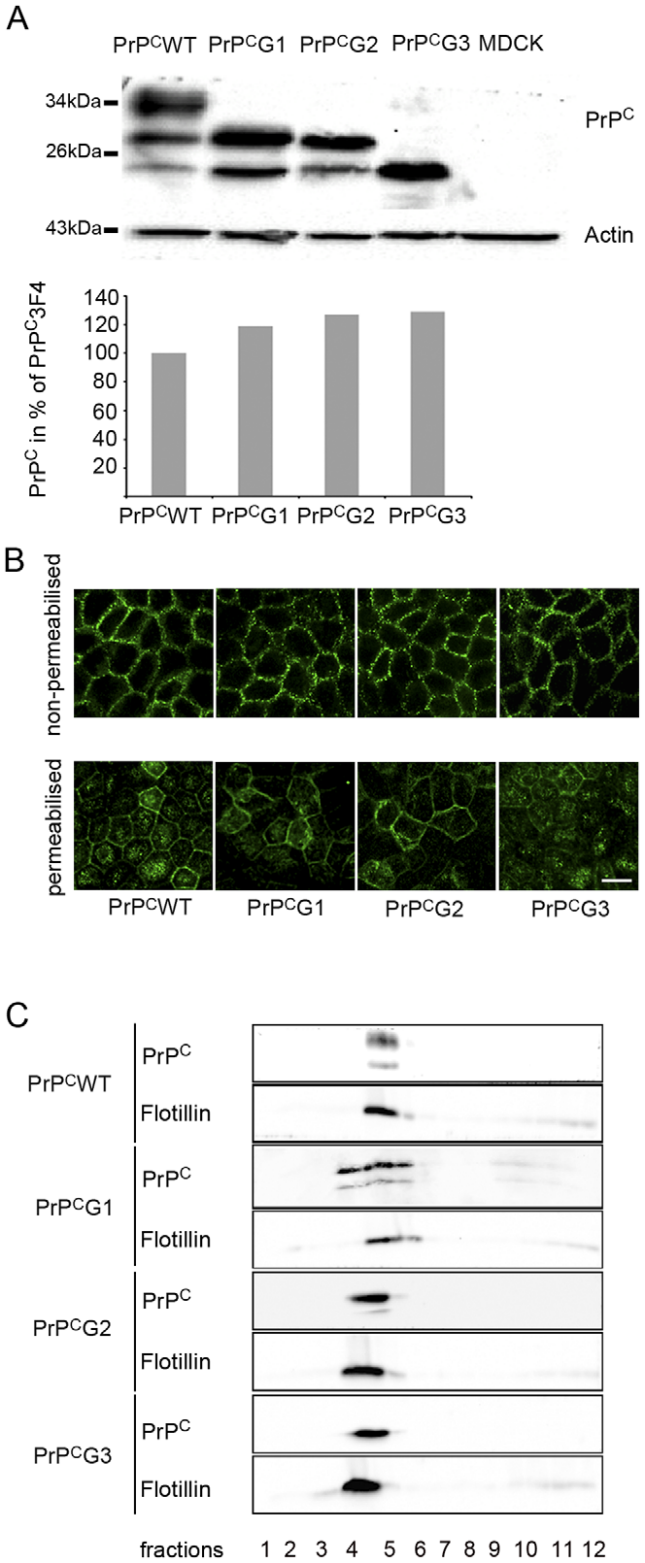
Physiological membrane localization of PrP^C^ glycomutants. (A) Characterization of glycomutants (PrP^C^G1, PrP^C^G2, and PrP^C^G3) and PrP^C^WT for the study by Western blot analysis, using an antibody directed against the 3F4 epitope. Clones with similar amounts of overexpressed 3F4 tagged PrP^C^ as assessed by densitometric analysis of Western blots were used for these analyses (see graph). Relative expression of various PrP^C^ forms is shown in percentages of PrP^C^WT that was set to 100%. (B) Assessment of plasma membrane (non-permeabilized) and intracellular (permeabilized) localization of PrP^C^ glycomutants by confocal microscopy shows presence of PrP^C^ at the plasma membrane and intracellularly (scale bar is 10 µm). (C) Assessment of DRMs localization of PrP^C^ glycomutants by Triton X-100 extraction at 4°C and sucrose density gradient centrifugation showing correct localization of PrP^C^ glycomutants with flotillin-positive DRM containing fractions.

One of the purposes of our study was to determine the role of the glycans in sorting of mouse PrP^C^. We therefore generated stably expressing PrP^C^ mutants in which the first (N180Q mutant, PrP^C^G1), the second (N196Q mutant, PrP^C^G2), and both (N180Q/N196Q mutant, PrP^C^G3) consensus sites for N-glycosylation were changed. The corresponding PrP^C^ represented mono- or non-glycosylated PrP^C^ in MDCK cells (Fig. 1). PrP^C^ glycomutants and wild-type PrP^C^ (PrP^C^WT) contained the 3F4 epitope tag allowing discrimination of overexpressed from endogenous PrP^C^ [32]. Clones with similar PrP^C^ expression levels were chosen for the study (Fig. 2A). Western blots showed that in extracts of PrP^C^WT expressing cells the typical PrP^C^ glycosylation pattern was detected with polypeptides of an approximate size of 34, 29, and 24 kDa. No immunoreaction was observed in non-transfected cells. PrP^C^G1 and G2 polypeptides showed two bands at 29 and 24 kDa whereas PrP^C^G3 represented a single band at 24 kDa.

In order to assess whether mouse PrP^C^ lacking N-glycans is correctly localized at the plasma membrane, we used confocal fluorescence microscopy of cells grown in Transwells. Under non-permeabilising conditions, PrP^C^G1, G2, G3, and PrP^C^WT were found to be present at the plasma membrane. When cells were permeabilised, non-glycosylated PrP^C^G3 showed the most intense intracellular labeling whereas PrP^C^G2 was mainly localized at the plasma membrane and PrP^C^G1 and PrP^C^WT could be found both at the plasma membrane and in intracellular membranes (Fig. 2B).

Since PrP^C^ is largely located in defined DRMs, we assessed distribution of PrP^C^WT and PrP^C^G1, G2, and G3 in insoluble fractions after Triton X-100 extraction and sucrose density gradient centrifugation. All glycomutants expressed in MDCK cells were correctly located in DRMs with patterns similar to PrP^C^WT (Fig. 2C).

### Monoglycosylated PrP^C^ sorts in an unpolarized manner in MDCK cells

**Figure 3:**
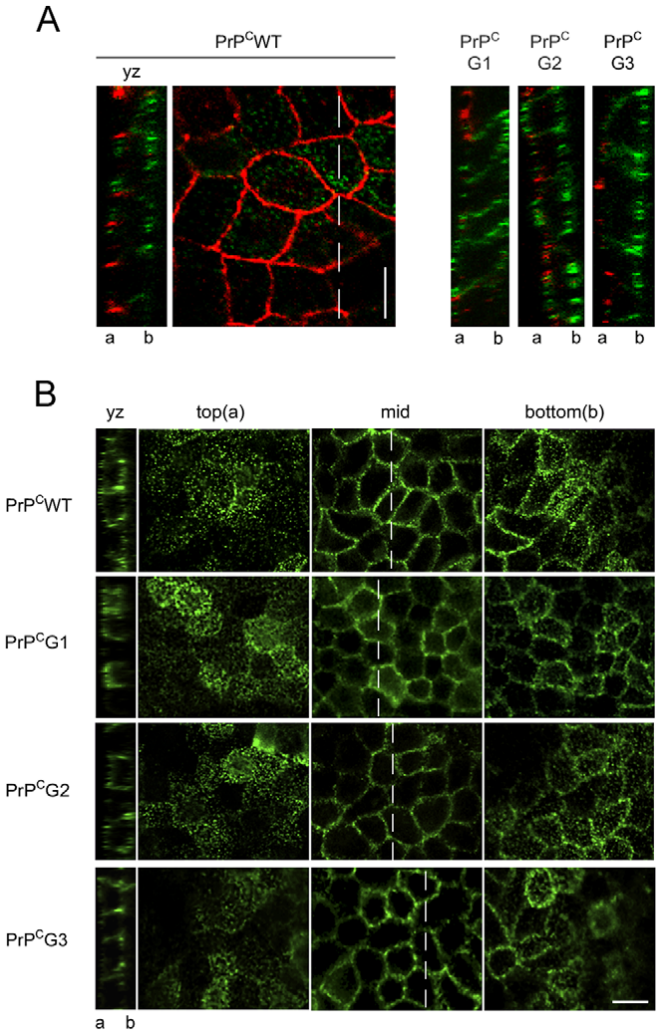
N-glycosylation of PrP^C^ affects polar sorting in MDCK cells. MDCK cells stably expressing PrP^C^WT, PrP^C^G1, PrP^C^G2 or PrP^C^G3 were grown in Transwells for 4 to 5 days until they were fully polarized. (A) Cells were separately stained with the 3F4 antibody (green) followed by permeabilisation and staining with an antibody against ZO-1 (red), a constituent of tight junctions, indicating the cell polarity. Confocal microscopy of a Z-stack of PrP^C^WT (left) at the level of tight junctions stained with ZO-1, and YZ-sections (right) of all glycomutants indicate both the integrity of the polarized monolayer and a redistribution of PrP^C^G1 and PrP^C^G2 to the apical compartment when compared to PrP^C^WT and PrP^C^G3. Localization of the apical (a) and basolateral (b) compartment is indicated. (B) After immunocytochemistry under non-permeabilising conditions with the 3F4 antibody, serial Z-stacks from the bottom to the top were taken with confocal microscopy. YZ images shows transversal cut trough cells at the mid level, marked with a dashed line. PrP^C^WT and PrP^C^G3 were mainly found in the basolateral compartment whereas PrP^C^G1 and PrP^C^G2 were mainly found in both compartments. Scale bars represent 10 µm.

To determine whether glycosylation affects the sorting of PrP^C^ in polarized cells, the expression at apical and basolateral membranes of mutant PrP^C^G1 to G3 in filter-grown MDCK cells was studied by immunofluorescence microscopy. ZO-1 antibody, labeling the tight junctions that separate apical from basolateral membrane [33], was used in order to verify full cell polarization (Fig. 3A). PrP^C^WT and PrP^C^G3 were mainly found in the basolateral compartment whereas PrP^C^G1 and PrP^C^G2 were found to be present both, in apical and the basolateral compartments (Fig. 3B).

**Figure 4:**
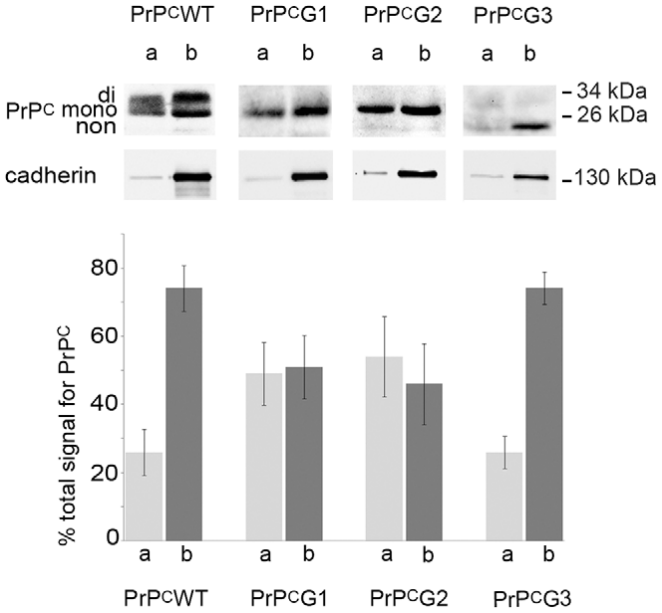
Cell surface biotinylation assay confirms a role of the N-glycans in polarized sorting of PrP^C^. Cells were grown in Transwells for 4–5 days until fully polarized and labelled with EZ-Link Sulfo-NHS-SS-Biotin either on the apical (a) or the basolateral (b) side. Cells were processed for PrP^C^ (recognized with the 3F4 antibody) and E-cadherin Western blotting in parallel. The graph indicates densitometric evaluation of Western blots of at least 3 independent experiments, expressed as mean percentages ± SEM apical (a) or basolateral (b) of total protein found, which is set at 100%.

In order to quantify PrP^C^ at the plasma membrane at steady state, cell surface biotinylation experiments of filter-grown MDCK cells were performed. E-cadherin served as a marker for the basolateral compartment [34] and was highly concentrated at the basolateral side (average of 94%, SEM ±0.76). Half of the total PrP^C^G1 and PrP^C^G2 were found at the apical and basolateral membrane (PrP^C^G1, 49%±7.6 basolateral, 51%±7.6 apical; PrP^C^G2 46%±11.8 basolateral, 54%±11.8 apical). PrP^C^G3 was enriched at the basolateral membrane, comparable to PrP^C^WT (PrP^C^WT, 74.1%±6.7 basolateral, 25.9%±6.7 apical; PrP^C^G3, 74%±4.8 basolateral, 26%±4.8 apical) (Fig. 4).

### Thy-1 GPI-anchor directs PrP^C^ to the apical membrane in MDCK cells

**Figure 5:**
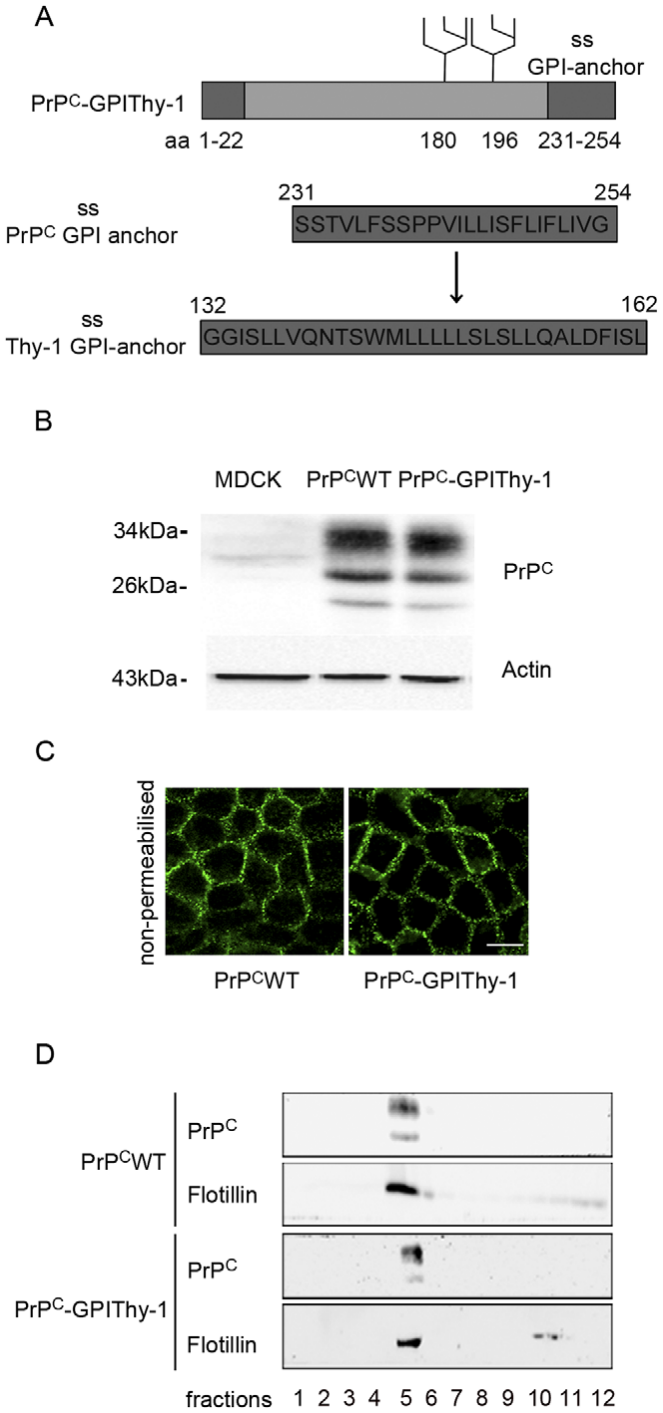
PrP^C^-GPIThy-1 is glycosylated and transported to the plasma membrane. (A) Schematic presentation of GPI-anchored PrP^C^WT and the PrP^C^ fusion protein with the GPI-anchor of Thy-1 (PrP^C^-GPIThy-1). The substitution of the GPI-anchor signal sequence (ss) of the PrP for the one of Thy-1 is indicated. (B) Western blots of PrP^C^WT and PrP^C^-GPIThy-1 stably expressed in MDCK cells. A clone with a similar expression level as PrP^C^WT was chosen. The glycotype of di-, mono-, and non-glycosylated PrP^C^-GPIThy-1 is unchanged. (C) Assessment of non-permeabilized membrane localization of PrP^C^WT and PrP^C^-GPIThy-1 by confocal microscopy shows plasma membrane localization of both proteins (scale bar is 10 µm). (D) Sucrose density gradient centrifugation of 1% Triton-X100 extraction at 4°C of PrP^C^WT and PrP^C^-GPIThy-1 cells reveal localization of both in flotillin enriched DRMs. Fractions were taken from the top (fraction 1) to the bottom (fraction 12).

PrP^C^ is a GPI-anchored protein. The fact that it is concentrated at the basolateral compartment in MDCK cells raises the question of the role of its GPI-anchor in sorting. Therefore, we stably expressed a fusion protein, comprising mouse PrP^C^ with the GPI-anchor signal sequence of Thy-1 (PrP^C^-GPIThy-1) in MDCK cells (Fig. 5A). Thy-1 is neuronally expressed and, like PrP^C^, found in DRMs but it is exclusively targeted to the apical compartment [35].

**Figure 6:**
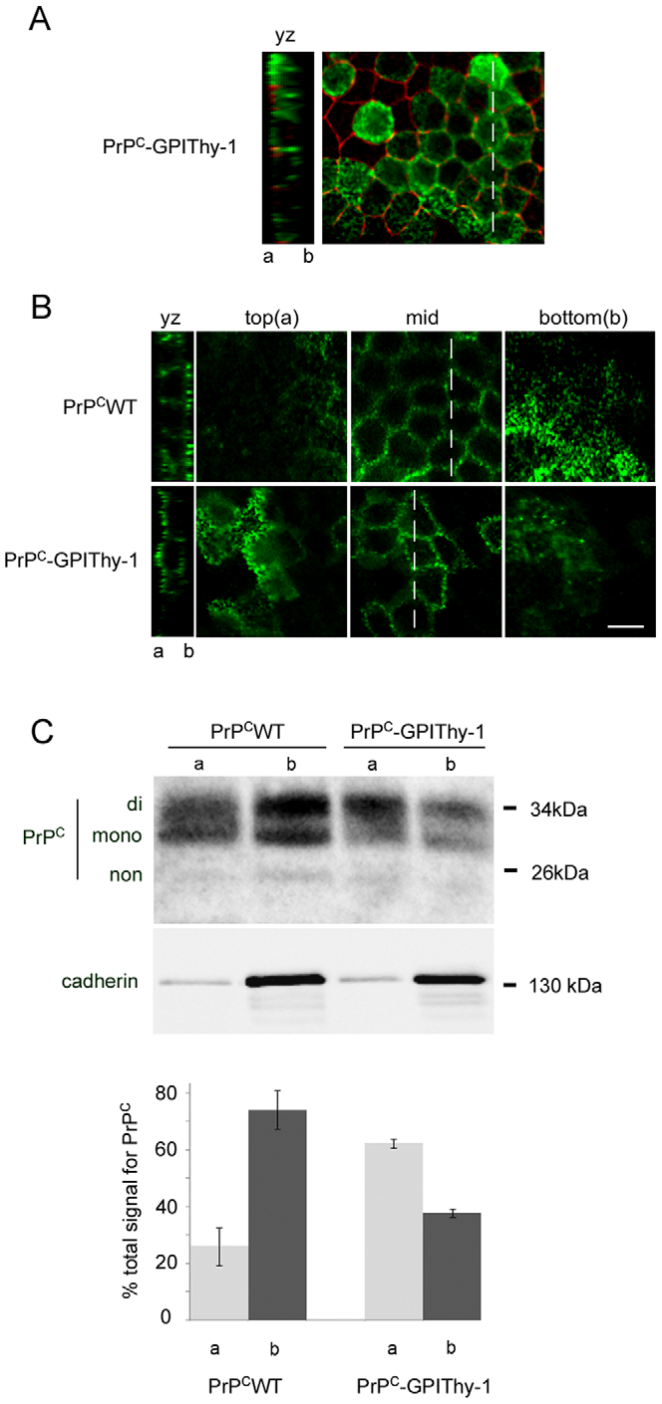
Thy-1-GPI anchor redirects PrP^C^ to the apical site. (A) Cells stably expressing PrP^C^WT and PrP^C^-GPIThy-1 were grown in Transwells for 4 to 5 days, processed for immunocytochemistry, and analyzed with confocal microscopy. YZ sections (left) and view on the membrane (right) at the level of tight junctions stained for ZO-1 (red) confirm both polarization and confluency of cells and show increased apical signal for PrP^C^-GPIThy-1 (green). (B) After staining with PrP 3F4 antibody under non-permeabilizing conditions, serial Z-stacks from the bottom to the top were taken. YZ sections show transversal cut through cells at the level of the dashed line in mid. PrP^C^-GPIThy-1 was found at the apical membrane when compared to PrP^C^WT. Scale bars are 10 µm. (C) Cells grown in Transwells labeled with EZ-Link Sulfo-NHS-SS-Biotin either apically (a) or basolaterally (b) were processed for Western blotting for PrP^C^ and E-Cadherin (as control of cell polarization) in parallel. The graph (three independent experiments) shows mean percentages ± SEM of apical (a) or basolateral (b) amount of protein when compared to the total amount which is set at 100%.

Cell clones with similar expression levels of PrP^C^ and PrP^C^-GPIThy-1 were chosen for further analysis (Fig. 5B). Western blot analysis revealed identical glycotypes of PrP^C^-GPIThy-1 and PrP^C^, with a prominent diglycosylated band in both cases. To exclude that the addition of the Thy-1 GPI-anchor affects intracellular transport, immunofluorescence microscopy under non-permeabilising conditions was performed. This showed localization and integration of PrP^C^-GPIThy-1 at the plasma membrane (Fig. 5D). In contrast to neurons, we could not detect shedded forms of PrP^C^ and PrP^C^-GPIThy-1 in the media (data not shown) indicating no substantial shedding in MDCK cells [36]. Triton X-100 extraction and sucrose density gradient centrifugation showed that PrP^C^-GPIThy-1, like PrP^C^, can be recovered in flotillin enriched DRM fractions (Fig. 5E). Confocal microscopy of cells grown in Transwells showed that PrP^C^-GPIThy-1 was mainly present in the apical compartment separated from the basolateral side by ZO-1 immunoreactive tight junctions (Fig. 6A and B). Cell surface biotinylation confirmed data of morphological analysis with PrP^C^-GPIThy-1 being mainly found in apical membranes (37.7%±1.5 basolateral, 62.3%±1.5 apical) (Fig. 6C).

## Discussion

In this study we investigated the role of the N-glycans and the GPI-anchor in polarized sorting of mouse PrP^C^ to gain deeper insight into the physiological function of PrP^C^ and into the pathophysiology of prion disease [11], [37], [38]. Under physiological conditions, the occupancy of the N-glycosylation sites at N180 and N196 of PrP^C^ is variable and cell dependent [39], [40]. In human brain, full length as well as truncated forms with variable glycosylation content are found [13] suggesting proper folding of all glycoforms [41].

Changes of Asn residues at codon 180 and 196 of PrP^C^ alter N-glycosylation without affecting cell surface expression of PrP^C^ or conversion to PrP^Sc^ [42], [43], whereas mutations of the Thr residues of the N-glycosylation consensus site Asn-X-Thr, that also eliminate the N-glycosylation, disturb intracellular trafficking [42], [43], [44], [45]. For our study we chose to eliminate N-glycosylation by substitution of Asn by Gln in both consensus sites (N180Q, N196Q) because our aim was to express all three glycoforms at the plasma membrane. All the glycomutants (N180Q, N196Q, and N180Q/ N196Q) used in our study were correctly inserted in lipid rafts at the plasma membrane. When permeabilised, however, we could observe an increased intracellular staining intensity for PrP^C^G3, indicating that non-glycosylated PrP^C^ is retained in intracellular membranes, most likely in the ER as previously described [41].

In our study we found that 74% of mouse PrP^C^ is present at the basolateral membrane of MDCK cells, in agreement with previous studies [26], [27]. Why human PrP^C^ is selectively targeted to the apical side in MDCK and Caco2 intestinal cells [46] is unclear. However, our data clearly show that the presence of only one N-glycan leads to unpolarized sorting whereas non-glycosylated mouse PrP^C^ is sorted, like wild-type PrP^C^, to the basolateral membrane. N-glycans have been postulated as one of the main apical targeting signals for polarized sorting of membrane proteins. Thus, GPI-anchored glycosylated membrane dipeptidase (MDP) is targeted to the apical membrane whereas the non-glycosylated MDP was found at the basolateral side [23]. Additionally, soluble proteins which are secreted in an unpolarized manner such as the rat growth hormone, are apically secreted after introduction of N-glycosylation sites [21]. Furthermore, the basolateral expression of Na,K ATPase B1 subunit in MDCK cells is altered and directed to the apical side after addition of mutagenesis-mediated N-glycosylation. The apical targeting is correlated to the extent of N-glycosylation [47].

Surprisingly, we found that the loss of one N-linked oligosaccharide either at N180 or N196 leads to an equal localization of PrP^C^ both at the apical and basolateral membrane in steady state. Similar observations have been reported by Sarnataro et al. [26] showing that the wild type PrP^C^ is transported first to both membrane sides of MDCK cells followed by accumulation of PrP^C^ at the basolateral membrane within 120 min. The authors explained the transient expression of PrP^C^ at the apical membrane by selective clearance or by internalization of apically expressed PrP^C^ and subsequent transcytosis to the basolateral side. Selective clearance or transcytosis are unlikely to explain the unpolarized distribution of monoglycosylated PrP^C^ because of the extracellular orientation of PrP^C^ oligosaccharide chains. Therefore, it is possible that the affinity or specificity of monoglycosylated PrP^C^ for binding to distinct lectins such as galectins 3 or 9 [48], [49] required for transport along the secretory pathway and/or sorting in the Golgi apparatus, are altered in comparison of wild type PrP^C^.

More than 30 different types of glycan chains have been identified by mass spectrometry to attach PrP^C^. Glycans attached at position N180 of mouse PrP^C^ have a lower proportion of tri- and tetra-antennary glycans and oligosaccharides at position N196 are more complex and acidic [50]. Molecular dynamic simulation of fully glycosylated human PrP^C^ showed that glycosylation at N181 plays a functional role, whereas glycosylation at N197, where the protein is more unstructured, plays a role in stabilization [51]. How the structure of monoglycosylated PrP^C^ is changed following the loss of one N-linked oligosaccharide, and its effect on lectin recognition in the ER deserves further studies.

Of interest, glycosylation patterns in the retina (comparable to the basolateral compartment) and the optic nerve (comparable to the apical compartment) differ in species with altered susceptibilty towards prion infection [52]. Recent data indicate that prion infection is a polarized event affected by glycosylation of PrP^C^. When PrP^C^ is not expressed in the compartment that is in contact with infectious prions, cells are not infected [53], [54]. Our data suggest that MDCK cells expressing mainly monoglycosylated PrP^C^ will be more prone to infection, due to the equal distribution in both the apical and basolateral compartment.

Furthermore, we show that the GPI-anchor functions as a strong polarity signal for PrP^C^. Chimeric PrP^C^-GPIThy-1 shows (i) a PrP^C^WT-like glycosylation pattern, (ii) an expression at the plasma membrane, and (iii) localization in DRMs. The redirection of PrP^C^-GPIThy-1 to the apical compartment, however, demonstrates the dominance of the GPI-anchor over N-glycosylation. At present, the molecular mechanism of sorting to different membranes between PrP^C^ and PrP^C^-GPI Thy-1 is unclear. It is known that the GPI-anchor affects protein structure and/or its interactions with the cell membrane [55]. In addition, the glycan moiety of the Thy-1 GPI-anchor that contains less complex sugar side chains than the PrP^C^ GPI-anchor, can occupy a carbohydrate binding site of the protein domain [56]. Finally, there are reports showing that although Thy-1 and PrP^C^ are DRM residents, they occupy domains that differ in their lipid composition [28], [57], [58]. The differential sorting can also be observed in neurons, where PrP^C^ is more enriched in the cell body and Thy-1 in neurites. [58]. Additionally, a hydrophobic core region in the ectodomain has been described that mediates basolateral sorting of PrP^C^ and leads to apical missorting upon site-directed mutagenesis [27]. These data and the results of our study indicate that PrP^C^ contains at least two independent signal structures, in the hydrophobic core and the GPI-anchor, directing PrP^C^ to the basolateral membrane. A third modulatory sorting motif is presented by the number of N-linked oligosaccharids in PrP^C^.
